# Supplementary figures and images for: Therapeutic Senolysis of Axitinib-Induced Senescent Human Lung Cancer Cells
Source: Cancers (Basel). 2024 Aug 7;16(16):2782. doi: 10.3390/cancers16162782 (PMC11352446; doi:10.3390/cancers16162782)

**Supplementary Figure S1.** The original Western blot.

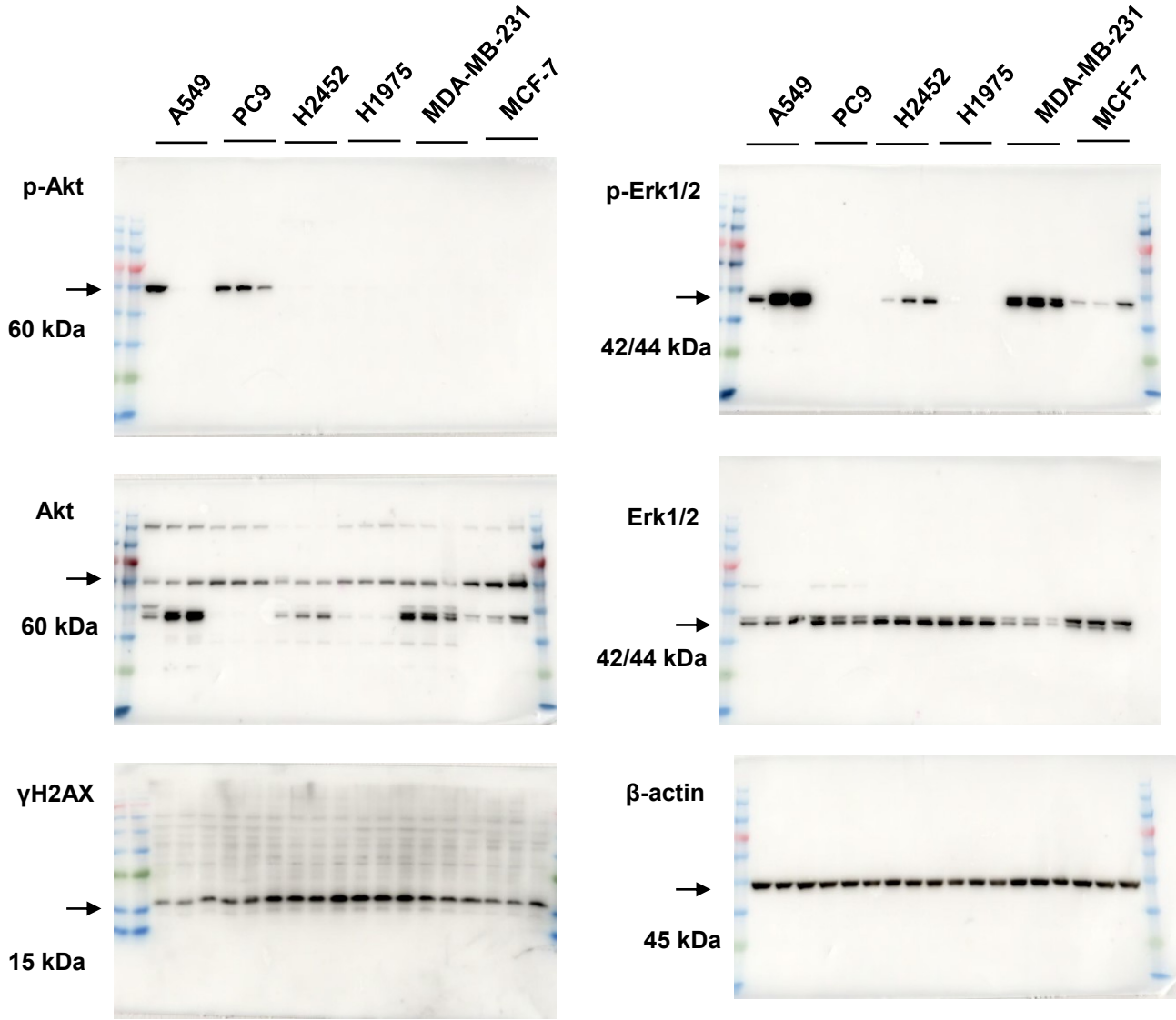

Supplement: Supplementary file 1 [file cancers-16-02782-s001.zip › cancers-3131450-supplementary.pdf]
